# In Support of “Propylene Glycol Toxicity in an Adolescent Secondary to Chronic Cornstarch Ingestion”

**DOI:** 10.1016/j.acepjo.2024.100007

**Published:** 2025-01-09

**Authors:** Kristen Downey, Ryan Keklik, Benjamin Morrissey, Robert Barnes, Karina Reyner, Michael Emmett

**Affiliations:** 1 Texas A&M College of Medicine, Dallas, Texas, USA; 2 Department of Emergency Medicine, Baylor University Medical Center, Dallas, Texas, USA; 3 Department of Internal Medicine, Baylor University Medical Center, Dallas, Texas, USA

To the Editor:

We thank Boshe et al^1^ for their interest in our recently published case report, “Propylene glycol toxicity in an adolescent secondary to chronic cornstarch ingestion” as they have raised several concerns about our diagnostic conclusions.^2^

The patient in our case study initially presented with an unexplained high anion gap—partially lactic—acidosis and severe acute kidney injury. Several hours after evaluation and empiric therapy, it was learned that she had been ingesting large quantities of corn starch every day. Soon after, her clinical status deteriorated, which resulted in profound metabolic acidosis, hyperosmolality, and multiorgan system failure. Peterson et al^3^ reported about a patient in whom a similar acute illness had developed after ingesting comparable amounts of daily corn starch; in that case, an osmolal gap and elevated levels of propylene glycol were documented. Unfortunately, in our case, the possibility of corn starch-related propylene glycol ingestion was not initially considered. Therefore, laboratory tests for measuring propylene glycol levels were not immediately ordered. With a half-life of 1.4 to 3.3 hours, the measurable level may have been metabolized to an undetectable level by the time of collection. However, we agree that our patient did not have a conclusive diagnosis of propylene glycol toxicity.

The main purpose of our report is to increase awareness of the possible relationship between chronic large-quantity corn starch ingestion and propylene glycol toxicity. We hope that increased awareness of this potential association will result in earlier consideration of this diagnosis so that appropriate studies can be performed. Time will tell if there is a real association between large amounts of daily corn starch ingestion and propylene glycol-related toxicity.

## Funding and Support

By *JACEP* *Open* policy, all authors are required to disclose any and all commercial, financial, and other relationships in any way related to the subject of this article as per ICMJE conflict of interest guidelines (see www.icmje.org). The authors have stated that no such relationships exist.

## Conflict of Interest

All authors have affirmed they have no conflicts of interest to declare.
